# Is the Effectiveness of Self-Visualization During Flexible Cystoscopy Gender-Dependent in Patients with no Previous Cystoscopy History? A Prospective Randomized Study

**DOI:** 10.1590/S1677-5538.IBJU.2024.0498

**Published:** 2025-01-05

**Authors:** Nurullah Hamidi, Mehmet Duvarci, Tuncel Uzel, Oguzhan Ceylan, Serhat Haluk Unal, Erdem Ozturk

**Affiliations:** 1 University of Health Science Dr. Abdurrahman Yurtaslan Ankara Oncology Training and Research Hospital Department of Urology Ankara Turkey Department of Urology, University of Health Science, Dr. Abdurrahman Yurtaslan Ankara Oncology Training and Research Hospital, Ankara, Turkey

**Keywords:** Anxiety, Cystoscopy, Patient Comfort

## Abstract

**Purpose:**

To evaluate the effect of real-time self-visualisation (SV) of the procedure during flexible cystoscopy (FC) on pain and anxiety in male and female patients with no prior cystoscopy history.

**Patients and Methods:**

Between Dec 2022-May 2024, 400 patients who underwent office-based FC were enrolled into prospective randomized study in accordance with CONSORT. Patients were randomised into two groups (SV and no-SV) using sequential (1:^1^ ratio) randomisation. To ensure equal numbers of male and female patients in each group, one consecutive male patient was assigned to the SV group, while the next male patient was assigned to the non-SV group; the same randomization was done for females. The primary endpoint was to evaluate the pain during FC (during urethral insertion of the cystoscope and bladder examination stages) of both groups. The secondary endpoint was to evaluate anxiety, patient satisfaction, and willingness to undergo the procedure of both groups.

**Results:**

In males, significant lower pain scores were detected in SV group during urethral insertion of the cystoscope (1.4 vs. 4.8, p<0.001) and during bladder examination (0.9 vs. 3.1, p<0.001). However, pain scores during urethral insertion of the cystoscope (1.9 vs. 2, p=0.38) and during bladder examination (1.2 vs. 1.3, p=0.63) were statistically similar between two groups in female patients. In both genders, significant lower anxiety levels, higher patient satisfaction and higher willingness to undergo repeat cystoscopy were detected in SV group.

**Conclusion:**

SV during FC may be beneficial in reducing pain in male patients but not in female patients. SV during FC has a positive effect on anxiety, patients’ satisfaction, and willingness to undergo repeat procedures, regardless of gender.

## INTRODUCTION

Cystoscopy is a procedure frequently performed by urologists in daily practice to diagnose various urological conditions such as bladder tumour (BT), benign prostatic hyperplasia, recurrent cystitis, and urethral stenosis. Cystoscopy can be performed in an operating room (under sedation, spinal block, or general anaesthesia) or as an office procedure (under local anaesthesia).

Office-based cystoscopies are important for reducing the workload in the operating room, especially for BT patients who require multiple cystoscopy procedures during follow-up each year. The procedure is usually well tolerated; however, it may cause mild to moderate pain, discomfort, and anxiety in some patients, even when a flexible cystoscope is used (1).

Experiencing pain during the procedure not only impacts the patient's quality of life but also affects the completion of the procedure. Patients may sometimes need to interrupt the procedure due to pain and postpone it to be performed under general anaesthesia. This can be particularly distressing for BT patients who require multiple cystoscopies throughout the year for follow-up. Pain and anxiety may even lead patients to consider skipping follow-up visits altogether.

Although pain relief methods such as intraurethral lidocaine-based lubricant application or distraction techniques like listening to music or watching relaxing videos during the procedure are used to alleviate pain and anxiety, an optimal solution has not yet been achieved (2, 3). In recent years, randomized controlled studies (RCSs) have also been published with conflicting results regarding the impact of patients watching their own cystoscopy video during the procedure as another distraction method (4-10). However, these studies evaluate mixed patient groups (male or female patients, first or repeat cystoscopy, cystoscopy alone or with additional procedures such as JJ stent removal) and have relatively small sample sizes. In some studies, most patients had a history of BT and thus had undergone at least one previous cystoscopy (4, 7, 9). It is well known that pain levels during the first cystoscopy are higher compared to repeated cystoscopies in BT patients undergoing surveillance (1).

We planned a randomised prospective study to evaluate the effect of real-time self-visualisation (SV) during flexible cystoscopy (FC) on pain and anxiety in male and female patients with no prior cystoscopy history. The rationale for selecting patients with no previous cystoscopy experience was to reveal the impact of real-time SV more clearly on pain and anxiety. Another distinction of our study from previous research is that we designed and managed it according to the Consolidated Standards of Reporting Trials (CONSORT) statement.

## MATERIAL AND METHODS

This prospective RCS was conducted at the outpatient clinic of our hospital after institutional ethical approval (Date:07.04.2021, Decision no:2021-04/1099). This trial was designed and managed based on the CONSORT guidelines (11, 12).

### Determination of sample size

A power analysis was conducted using the G*Power (v3.1.9.6) software to determine the sample size. The sample size for the study was calculated to achieve a power of 95%, with a significance level set at 0.05. Estimated pain scores were based on the mean pain values [mean VAS (Visual analogue scale) scores were 1.66±1.4 and 4.39±2.4 in SV and no-SV groups] reported in Soomro et al.'s study (8). Consequently, a total of 210 volunteers were required, comprising 105 volunteers in each group. We aimed to recruit 200 volunteers in each group to avoid potential volunteer dropouts and statistical errors.

### Patients, inclusion, and exclusion criteria

**Figure 1:**
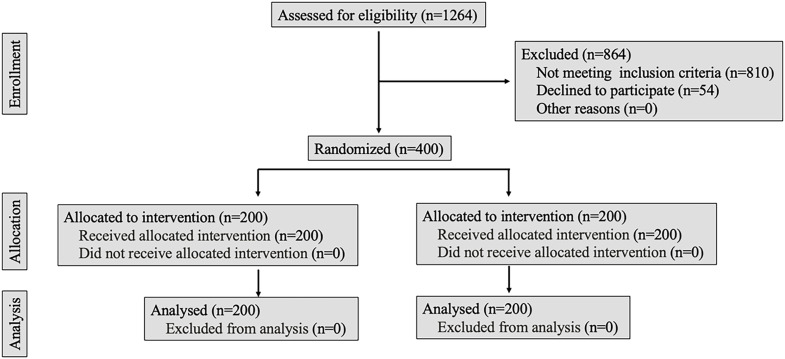
Flowchart of the study.

Four hundred patients (aged 18 or older) who underwent office-based FC were included in the study between Dec 2022 and May 2024. Indications for cystoscopy were haematuria (suspicious of BT), lower urinary tract symptoms (LUTS), and incontinence. Patients with a history of cystoscopy, active urinary tract infection, those who could not communicate, understand written material, or complete forms independently were excluded from the study. Additionally, patients with psychiatric disorders, language barriers, or a history of urethral stenosis (detected during the procedure) were not included. Patients requiring other procedures during FC, such as biopsy, fulguration for superficial BT, urethral dilation, or removal of foreign bodies or JJ stents, were also excluded from the study. The study design is summarized in the CONSORT flow chart (Figure-1).

### Randomization

Patients were randomized into two groups using sequential (1:^1^ ratio) randomization. To ensure an equal number of male and female patients in each group, one consecutive male patient was assigned to the SV group, while the next male patient was assigned to the no-SV group. Similarly, one consecutive female patient was assigned to the SV group, and the next female patient was assigned to the no-SV group.

### Cystoscopy procedure

Before the procedure, patients were positioned in the lithotomy position. After scrubbing with an iodine-based solution and standard draping, local anesthesia consisting of 2% lidocaine gel was applied to the urethra. A 15F flexible cystoscope (Fiber-Cystoscope WL40, Richard-Wolf) was then inserted through the urethral meatus. All procedures were performed by final-year residents (MD, OC). For patients in the SV group, a monitor was positioned for both the urologist and the patient. For patients in the no-SV group, the monitor was positioned only for the urologist.

The primary endpoint was to evaluate the pain during FC in both groups. The secondary endpoint was to evaluate anxiety, patient satisfaction, and willingness to undergo the procedure in both groups.

### Pain, satisfaction, and willingness to undergo the procedure evaluations

The pain was assessed during the passage of the cystoscope through the urethra and during the bladder examination. Pain levels were quantified using a VAS ranging from 0 to 10, with higher scores indicating greater pain. Additionally, patient satisfaction and willingness to undergo repeat cystoscopy (if needed) were evaluated using the VAS by a nurse who was blinded to the study protocol after the patient had dressed.

### Anxiety evaluations

Before (after the patient was informed about the procedure and possible complications) and immediately after the cystoscopy, anxiety levels were evaluated using the State Trait Anxiety Inventory (STAI) by a nurse who was blinded to the study protocol. A self-reported anxiety inventory comprised 20 questions. STAI scores range from 20 to 80, with higher scores indicating greater anxiety levels.

### Statistical Analysis

Statistical analysis was performed using SPSS version 25 (IBM, Chicago, IL, USA). The normality of the data was assessed using the Shapiro-Wilk (W) Test. Qualitative variables such as gender and indications for cystoscopy were considered categorical. The Chi-square test was used to compare categorical variables, which were expressed as counts and percentages. Quantitative variables, such as age, VAS scores, STAI scores, heart rates, and systolic blood pressure values, were expressed as mean and standard deviation. Student t-test was employed to compare independent groups of quantitative variables. The p-value of < 0.05 was considered statistically significant.

## RESULTS

### General data and comparison of all patients according to SV status without gender distribution

Four hundred patients (136 female and 264 male) were randomized into two groups (no-SV and SV groups), taking into account gender distribution. The mean age of the patients was 57.4±13.6 years. Indications for cystoscopy were haematuria, LUTS and incontinence in 292(73%), 67(16.7%), and 41(10.3%) patients, respectively. BT was revealed during FC in 76 (19 %) patients. The mean pain scores on VAS during urethral insertion of the cystoscope and during bladder examination were 2.76±2 and 1.79±1.5, respectively. Pre-cystoscopy and post-cystoscopy anxiety levels on STAI were 50.4±17.6 and 35.2±13.5, respectively. Post-cystoscopy patients’ satisfaction and willingness to undergo repeat cystoscopy levels on VAS were 6.2±1.8 and 6.1±2, respectively (Table-1).

**Table 1:** Comparison of patients according to self-visualization status in all patients without gender distribution.

Variables		All patients (n=400)	no-SV (n=200)	SV (n=200)	p value
Age (Years), Mean ± SD		57.4±13.6	57.9±13.3	57±14	0.48
**Gender, n (%)**					-
	Female	136 (34)	68 (34)	68 (34)	
	Male	264 (66)	132 (66)	132 (66)	
**Indication for cystoscopy, n (%)**					0.49
	Haematuria	292 (73)	150 (75)	142 (71)	
	LUTS	67 (16.7)	29 (14.5)	38 (19)	
	Incontinence	41 (10.3)	21 (10.5)	20 (10)	
Presence of BT at cystoscopy, n (%)		76 (19)	37 (18.5)	39 (19.5)	0.8
Pre-cystoscopy heart rate (beats/min), Mean ± SD		69.2±11.3	69±11.2	69.3±11.4	0.75
Post-cystoscopy heart rate (beats/min), Mean ± SD		78.9±13.9	82.3±13.7	75.5±13.3	<0.001*
Pre-cystoscopy systolic blood pressure (mmHg), Mean ± SD		119±12.5	118.3±12.7	119.8±12.3	0.24
Post-cystoscopy systolic blood pressure (mmHg), Mean ± SD		124.3±19.2	124.2±19.1	124.3±19.4	0.95
Pain during urethral insertion of the cystoscope (VAS), Mean ± SD		2.76±2	3.9±2.1	1.6±1.1	<0.001*
Pain during bladder examination (VAS), Mean ± SD		1.79±1.5	2.53±1.8	1.04±0.7	<0.001*
Pre-cystoscopy anxiety (STAI), Mean ± SD		50.4±17.6	49.6±17.3	51.1±17.9	0.38
Post-cystoscopy anxiety (STAI), Mean ± SD		35.2±13.5	41.9±14.5	28.4±7.7	<0.001*
Patients’ satisfaction (VAS), Mean ± SD		6.2±1.8	4.9±1.3	7.6±1.03	<0.001*
Willingness to undergo repeat cystoscopy (VAS), Mean ± SD		6.1±2	4.5±1.6	7.6±1.05	<0.001*

BT = Bladder tumour; LUTS = Lower urinary tract symptoms; SD = Standard Deviation; STAI = State Trait Anxiety Inventory; SV = Self-visualization; VAS = Visual analog scale

* Statistically significant

There were no significant differences between no-SV and SV groups in terms of patient age, indications, presence of BT, pre-cystoscopy heart rate, pre-cystoscopy systolic blood pressure, post-cystoscopy systolic blood pressure, pre-cystoscopy anxiety levels. Post-cystoscopy heart rate, pain during urethral insertion of the cystoscope, pain during bladder examination and post-cystoscopy anxiety levels were significantly lower in SV group compared with those in no-SV group (for all comparisons p<0.001). Patients’ satisfaction and willingness to undergo repeat cystoscopy were statistically significant higher in SV group than no-SV group (for all comparisons p<0.001). Comparisons between no-SV and SV groups are detailed in Table-1.

### Comparison of female patients according to SV status

Post-cystoscopy heart rate was significantly lower in SV group than no-SV group (p<0.001). No statistically significant effect of SV on pain levels in both stages (during urethral insertion of the cystoscope and bladder examination) of FC was detected in female patients (p>0.05). However, post-cystoscopy anxiety levels were statistically significant lower in female patients who underwent SV(p<0.001). Post-cystoscopy patients’ satisfaction and willingness to undergo repeat cystoscopy were statistically significant higher in SV group than no-SV group (for all comparisons p<0.001). Other variables were comparable in SV and no-SV groups. Comparisons of female patients are detailed in Table-2.

**Table 2:** Comparison of female patients according to self-visualization status.

Variables		Female patients (n=136)	no-SV (n=68)	SV (n=68)	p value
Age (Years), Mean ± SD		55 ± 13.6	56.6 ± 13.3	53.4 ± 13.7	0.17
**Indication for cystoscopy, n (%)**					0.6
	Haematuria	94 (69.1)	47 (69.1)	47 (69.1)	
	LUTS	13 (9.6)	5 (7.4)	8 (11.8)	
	Incontinence	29 (21.3)	16 (23.5)	13 (19.1)	
Presence of BT at cystoscopy, n (%)		20 (14.7)	12 (17.6)	8 (11.8)	0.33
Pre-cystoscopy heart rate (beats/min), Mean ± SD		68.5±11.5	68.7±11.2	68.3±11.9	0.87
Post-cystoscopy heart rate (beats/min), Mean ± SD		80.1±14.5	84.2±14	76±13.8	0.001*
Pre-cystoscopy systolic blood pressure (mmHg), Mean ± SD		118.7±11.6	117.6±12	119.8±11.1	0.26
Post-cystoscopy systolic blood pressure (mmHg), Mean ± SD		124.5±18.6	122.1±19.3	126.9±17.7	0.13
Pain during urethral insertion of the cystoscope (VAS), Mean±SD		1.95±0.9	2±0.9	1.9±0.8	0.38
Pain during bladder examination (VAS), Mean± SD		1.29±0.7	1.32±0.7	1.26±0.7	0.63
Pre-cystoscopy anxiety (STAI), Mean ± SD		52.3±16.7	51.8±16.3	52.8±17.2	0.72
Post-cystoscopy anxiety (STAI), Mean ± SD		36.4±13.3	43.9±13.4	28.7±7.7	<0.001*
Patients’ satisfaction (VAS), Mean ± SD		6.2±1.8	4.7±1.2	7.6±1	<0.001*
Willingness (VAS), Mean ± SD		6±2.1	4.4±1.7	7.5±1	<0.001*

BT = Bladder tumour; LUTS = Lower urinary tract symptoms; SD = Standard Deviation; STAI = State Trait Anxiety Inventory; SV = Self-visualization; VAS = Visual analog scale

* Statistically significant

### Comparison of male patients according to self-visualization status

Post-cystoscopy heart rate was significantly lower in SV group than no-SV group (p<0.001). SV was determined to have a statistically significant positive effect on pain during urethral insertion of the cystoscope, bladder examination stages and post-cystoscopy anxiety levels in male patients (for all comparisons p<0.001). Moreover, post-cystoscopy patients’ satisfaction and willingness to undergo repeat cystoscopy were statistically significant higher in SV group than no-SV group (for all comparisons p<0.001). Other variables were comparable in SV and no-SV groups. Comparisons of male patients are detailed in Table-3.

**Table 3:** Comparison of male patients according to self-visualization status.

Variables		Male patients (n=264)	no-SV (n=132)	SV (n=132)	p value
Age (Years), Mean ± SD		58.6 ± 13.6	58.5 ± 13.2	58.7 ± 14	0.9
**Indication for cystoscopy, n (%)**					0.51
	Haematuria	198 (75)	103 (78)	95 (72)	
	LUTS	54 (20.5)	24 (18.2)	30 (22.7)	
	Incontinence	12 (4.5)	5 (3.8)	7 (5.3)	
Presence of BT at cystoscopy, n (%)		56 (21.2)	25 (18.9)	31 (23.5)	0.45
Pre-cystoscopy heart rate (beats/min), Mean ± SD		69.5±11.2	69.2±11.2	69.9±11.1	0.61
Post-cystoscopy heart rate (beats/min), Mean ± SD		78.3±13.6	81.3±13.5	75.3±13	<0.001*
Pre-cystoscopy systolic blood pressure (mmHg), Mean ± SD		119.2±13	118.7±13.1	119.8±13	0.51
Post-cystoscopy systolic blood pressure (mmHg), Mean ± SD		124.1±19.5	125.3±19	123±20	0.34
Pain during urethral insertion of the cystoscope (VAS), Mean ± SD		3.2±2.2	4.8±1.8	1.4±1.1	<0.001*
Pain during bladder examination (VAS), Mean ± SD		2.1±1.8	3.1±1.8	0.9±0.7	<0.001*
Pre-cystoscopy anxiety (STAI), Mean ± SD		49.3±18	48.4±17.8	50.2±18.2	0.42
Post-cystoscopy anxiety (STAI), Mean ± SD		34.5±13.5	41±15.1	28.1±7.6	<0.001*
Patients’ satisfaction (VAS), Mean ± SD		6.3±1.7	4.9±1.3	7.6±1	<0.001*
Willingness (VAS), Mean ± SD		6.1±2	4.6±1.6	7.6±1	<0.001*

BT = Bladder tumour; LUTS = Lower urinary tract symptoms; SD = Standard Deviation; STAI = State Trait Anxiety Inventory; SV = Self-visualization; VAS = Visual analog scale

* Statistically significant

## DISCUSSION

In this RCS, we observed that SV during FC significantly reduced pain in male patients, although this effect was not observed in female patients. Additionally, SV during FC was found to have a positive impact on anxiety in both genders. A key difference between our study and previous studies is the exclusion of patients with prior cystoscopy experience, allowing us to more accurately assess the effect of SV on pain and anxiety. Another distinguishing feature of our study is that it was designed and conducted in accordance with the CONSORT statement. We also found that SV has a clear positive effect on patient satisfaction and their willingness to undergo repeat procedures. Importantly, our study is the first to address these specific outcomes, as no prior data exists on the impact of SV on patient satisfaction or willingness to repeat procedures.

To date, several RCSs (4-10) have been published on the effect of SV of pain during FC, with varying outcomes. Firstly, Clements et al. reported an RCS involving 129 patients (4). They evaluated pain at the different stages of the procedure (during insertion of the scope and bladder examination). Pain levels were classified as none (VAS score 1), mild (VAS score 2-3), moderate (VAS score 4-6) and severe (VAS score 7-10) degrees. They reported that video viewing had an effect on pain during bladder examination (p=0.028) whereas it had no effect during scope insertion (p=0.79). They also evaluated anxiety levels using a four-point descriptive scale (none, mild, moderate, and severe) and found no significant difference between groups (p=0.189). There were some methodological concerns. Firstly, no data on the gender distribution of the participants was provided. Secondly, instead of comparing mean VAS scores for pain, pain levels were categorized by severity, with a percentage comparison made. Additionally, the objectivity of anxiety levels using a four points scale is questionable. Nowadays, anxiety levels can be measured more objectively with scales such as STAI or Beck Anxiety Inventory scores.

Patel et al. published their outcomes for both genders in two different RCSs (5, 6). First, they evaluated the effect of SV on pain in 100 male patients who underwent FC and determined that the mean VAS (evaluated with a 100 mm unmarked horizontal line) score was statistically lower in the SV group than no-SV group (14 vs. 23, p=0.02) (5). One year later, the effect of SV on pain was evaluated in a 100 female population who underwent rigid cystoscopy (6). However, they could not demonstrate the positive effect of SV on reducing pain in the female population (6).

In another study, 114 patients were randomized to SV and no-SV groups (7). They detected statistically similar pain levels into two groups (p=0.18) (7). In the same study, they also subdivided all patients according to their cystoscopy history and compared pain levels. They revealed that there was no statistically significant effect of SV on pain in patients who had first cystoscopy (p=0.23), who had 2-5 cystoscopies (p=0.58), and who had > 5 cystoscopies (p=0.37) (7).

Two low-sample size RCS have been published showing that SV during FC is effective in reducing pain in male patients (8,9). Somro et al. evaluated 76 patients and they found patients who viewed their FC had lower VAS scores (1.66 vs. 4.39, p<0.001) (8). Somro et al. highlighted that the different results obtained compared to previous studies (the positive effect of SV on pain) might be attributed to differences in the patient's position and geographical location (8). They conducted all procedures in supine position, unlike previous studies. The supine position may make patients feel more comfortable than the lithotomy position, which could help prevent pain. However, we attribute this difference to the characteristics of the patients, as in Somro et al. study (8), 42% of the patients underwent additional invasive and potentially painful procedures (like JJ stent removal).

In the second RCS analysed, Zhang et al. (9) included only male patients and excluded patients who underwent additional procedures during FC, like our study. However, they included patients undergoing first or repeated cystoscopy. Pain levels were lower in patients who watched their procedure (1.12 vs. 3.33, p<0.001) (9). They also divided the patients into subgroups (first cystoscopy, previous cystoscopy history, diagnostic cystoscopy, surveillance cystoscopy for BT). It was shown that watching the procedure is statistically significantly beneficial in reducing pain across all patient subgroups (9).

In the present study, we revealed that SV of the FC significantly reduced pain in male patients, though this effect was not reached in female patients. Additionally, we found that men experienced more pain than women during both stages of the cystoscopy procedure. We believe this difference between genders is due to anatomical differences. Urethral length is short in females, making them less likely to experience pain during insertion of the cystoscope. Moreover, elongation in the craniocaudal diameter due to prostate enlargement, particularly in older men, and narrowing at the membranous urethra level may cause increased pain. Taghizadeh et al. (13) and Chen et al. (14) noted that the most painful part of the procedure occurs when the tip of the flexible cystoscope passes through the membranous urethra in men. For these reasons, it is expected that females experience less pain and discomfort during FC procedures compared to males.

More recently, González-Padilla et al. (10) evaluated the impact of SV of the procedure on pain in 318 male and 86 female patients. In this quasi-randomized study, they found a beneficial effect of SV on pain (VAS scores) in female patients (1.64 vs. 2.78, p=0.008) but not in male patients (2.5 vs. 2.6, p=0.276) (10). This difference between genders can be attributed to some reasons. González-Padilla et al. (10) used flexible and rigid cystoscopes during cystoscopy procedures in male and female patients, respectively. The effect of the flexible cystoscope on pain and patient comfort is well known (15). Therefore, SV may not make much difference on pain in patients who are undergoing FC. However, SV may have made a difference in patients who were undergoing rigid cystoscopy. To avoid this controversy, we used flexible cystoscopes for both genders in our study.

Moreover, González-Padilla et al. (10) stated that conducting their study using a quasi-randomized method due to logistical constraints may have introduced some theoretical weaknesses. There is another detail in this study despite existing theoretical weaknesses and the conclusion that the SV of the procedure only reduces pain in females. They emphasized that the number of previous cystoscopies has an influence, diminishing the perception of pain, regardless of whether the patient visualizes the procedure or not. They reported a higher mean VAS score in patients who have no cystoscopy history than patients who have three or more cystoscopy histories in males (3.1 vs. 2.1, p=0.001) and in females (2.89 vs. 1.56, p=0.02) (10). These findings have been also supported by data from 1320 consecutive cystoscopies showing that pain level during the first cystoscopy is higher than for repeated cystoscopies (1). Therefore, while planning our study, we excluded patients with a previous history of cystoscopy to evaluate the effect of SV on pain more clearly. Patients who have had cystoscopy more than once usually feel less pain as they become accustomed to the procedure. Additionally, we excluded from the study patients who would require additional procedures such as JJ stent removal, biopsy, and stenosis dilation, which would prolong the procedure time and therefore probably make the patient feel more pain.

Anxiety levels can vary between individuals, regardless of SV. Waiting for the results of a diagnostic procedure can be a significant source of anxiety. Real-time information about a "normal" examination may help reduce this anxiety. However, the bias towards potential malignancy could be related to the absence or presence of abnormal findings during the diagnostic procedure, rather than the SV. Therefore, we also analysed the cystoscopy findings of the patients included in the study. We found no differences in the rates of malignancy detected during cystoscopy between the SV and no-SV groups for both genders.

Although our sample size is large and our methodology was well-planned, there are some limitations. We performed all procedures in the lithotomy position for both genders. Literature suggests that patients may be more comfortable during cystoscopies performed in the supine position rather than lithotomy (8). FC under the supine position could be considered for female patients, but we were unable to implement this due to the specific design of the patient bed in the outpatient clinic, which is tailored for the lithotomy position.

## CONCLUSIONS

In conclusion, SV during FC may be beneficial in reducing pain in male patients but not in female patients. SV during FC has a positive effect on anxiety, patients’ satisfaction, and willingness to undergo repeat procedures, regardless of gender.

## Data Availability

Due to personal data security laws, the data sets created and/or analysed for this study are not available to the public. However, they can be obtained from the associated author upon reasonable request.

## ABBREVIATIONS

BT: - Bladder Tumour
FC: - Flexible Cystoscopy
LUTS: - Lower Urinary Tract Symptoms
RCS: - Randomized Controlled Study
SV: - Self-Visualisation
STAI: - State Trait Anxiety Inventory
VAS: - Visual Analogue Scale
